# Health Effects of Alternate-Day Fasting in Adults: A Systematic Review and Meta-Analysis

**DOI:** 10.3389/fnut.2020.586036

**Published:** 2020-11-24

**Authors:** Yuanshan Cui, Tong Cai, Zhongbao Zhou, Yingmei Mu, Youyi Lu, Zhenli Gao, Jitao Wu, Yong Zhang

**Affiliations:** ^1^Department of Urology, The Affiliated Yantai Yuhuangding Hospital of Qingdao University, Yantai, China; ^2^Department of Urology, Beijing Tian Tan Hospital, Capital Medical University, Beijing, China; ^3^Binzhou Medical University, Yantai, China; ^4^Department of Allergy, The Affiliated Yantai Yuhuangding Hospital of Qingdao University, Yantai, China

**Keywords:** alternate day fasting, calorie restriction (CR), body weight, weight loss, meta-analysis, randomized controlled trials (RCT)

## Abstract

**Background:** Alternate-day fasting (ADF) method is becoming more and more popular among adults. This meta-analysis aims to evaluate the effects of ADF on adults.

**Methods:** Randomized controlled trials (RCTs) of ADF were searched using PubMed (1988 to March 2020), EMBASE (1995 to March 2020), and the Cochrane Controlled Trials Register. A systematic review was carried out using the Preferred Reporting Items for Systematic Reviews and Meta-analyses. The datum was calculated by RevMan version 5.3.0. The original references for relating articles were also reviewed.

**Results:** Seven randomized controlled trials involving 269 participants (152 in the ADF group and 117 in the control group) were studied. In this meta-analysis, compared with the control group, the ADF group showed statistically significant reductions in weight (*p* < 0.00001) and body mass index (*p* < 0.00001). Besides, the ADF group showed significant differences in terms of total cholesterol (*p* = 0.001), low-density lipoprotein (*p* = 0.01), triglycerides (*p* = 0.02), fat mass (*p* = 0.002), lean mass (*p* = 0.002), systolic blood pressure (*p* = 0.003), diastolic blood pressure (*p* = 0.007), and total calorie intake (*p* = 0.007). At the same time, the analysis demonstrated that the ADF group had a same effect compared with control group in aspects of high-density lipoprotein (*p* = 0.27), homeostasis model assessment-insulin resistance (*p* = 0.55), and fasting blood sugar (*p* = 0.09).

**Conclusions:** This meta-analysis suggests that ADF is a viable diet strategy for weight loss, and it has a substantial improvement in risk indicators for diseases in obese or normal people.

## Introduction

It is well-established that obesity is a risk factor in some metabolic diseases, such as atherosclerosis, dyslipidemia, and nonalcoholic fatty liver disease, and the factor could be modified (1). The energy imbalance caused by increased calorie intake and decreased physical activity is an important reason for adult obesity (2). Among them, the long-term excessive carbohydrate intake has the most serious negative impact on the human body (3). Therefore, restricting calorie intake and increasing energy consumption are among the main methods to manage obesity. Meanwhile, people are investigating many manners of diet and exercise continuously for obese and overweight individuals (4–9). Some data showed that dietary restriction and physical exercise could maintain lean and weight loss. Of course, restrictive diets alone can also reduce lean body weight (4, 10, 11). Daily calorie restriction therapy is one of the most widely used diets for obese patients. This method can reduce energy intake by 15–40% every day (12, 13). Many people have lost weight successfully by using daily calorie restriction regimens; however, many obese people find it is difficult to insist on this approach because food intake must be limited every day. So, traditional daily calorie restriction therapy has poor compliance and long-term compliance (14, 15).

A recent study showed that caloric restriction could extend lifespan in rhesus monkeys (16, 17). In clinical trials, calorie restriction could reduce the risk factors of diseases and improve patient's health. However, a link has been found between continuous caloric restriction and harmful factors about human health (18). Overall, calorie restriction has many limitations, particularly on healthy humans. Alternate-day fasting (ADF), as a new calorie restriction method for obesity patients, has been proved to improve human health-related outcomes (9, 19–21). ADF involves a “fast day” where individuals consume 25% of energy needed, alternated with a “feed day” where subjects eat *ad libitum*, with days of eating freely and to appetite (*ad libitum*) (22). ADF may be an effective alternative for weight loss because this diet only requires calorie counting every other day (21, 23). In long-term human trials (2–3 months), ADF apparently lost body weight by about 3–7% and decreased the risk of artery disease and other biochemical indexes of the human body [low-density lipoprotein (LDL) cholesterol, triglycerides (TGs), and so on] (19, 24, 25).

According to relevant reports, obese patients with 25% calorie intake on fast days have been proved to be safe for at least 2 months; the characteristics of few adverse incidences have also been recognized by many people (26). In principle, some reports showed that adverse effects of ADF were minimal, such as mild headache or substantial hunger and light-headedness (27).

The ADF method has gotten more and more popular in the past decade. Some books of ADF became a bestseller; to some extent, these books also promote the popularity of weight-loss methods (28). Hitherto, it has been published more than a million copies in the United States and the United Kingdom (29). The body weight fluctuations made a great influence on people's physiology indexes. To evaluate the effects of ADF for adults, we performed a systematic review and meta-analysis of randomized controlled trials (RCTs).

## Materials and Methods

### Study Design

A systematic review of RCTs was carried out using the Preferred Reporting Items for Systematic Reviews and Meta-Analyses checklist (30).

### Search Strategy

Our study searched PubMed (1988 to March 2020), EMBASE (1995 to March 2020), and the Cochrane Controlled Trials Register, looking for studies about the effect of ADF on the metabolism of the human body. The keywords and medical subjects are as follows: “alternate day fasting,” “calorie restriction,” “body weight,” “weight loss,” “obesity,” “non-obese humans,” “exercise,” “fasting,” “meal replacement,” and “RCT.” If necessary, we also contacted the author to obtain more information about their research. Furthermore, we also reviewed original references for including texts.

### Inclusion and Exclusion Criteria

RCTs that met the following criteria were included: (a) the studies should have a connection with the topic: the effect of ADF on the metabolism of the human body (obese or nonobese humans), (b) full text of the study could be provided, or (c) precise data could be extracted, and there are similar indicators between the ADF group and the control group in every RCT.

The following studies were excluded: (a) not RCT, such as abstract, review, or comment or (b) incomplete data study.

Furthermore, we included the most recently published study if studies described identical experiments. Besides, every study would be included if different measures were evaluated. Table 1 shows the specific inclusion and exclusion criteria.

**Table 1:** Criteria for considering studies for the review based on the population, intervention, comparator, outcomes, and study designs (PICOS) structure.

	**Population**	**Intervention**	**Comparator**	**Outcomes**	**Study designs**
Inclusion criteria	Patients with BMI more than 17.5 kg/m^2^	ADF (4:3):25–30% of daily recommend energy intake or refrain from calorie completely on fast days	Controls:Maintain their present lifestyle.	Weight, BMI, TC, LDL, TG, HDL, FBS, fat mass, lean mass, SBP, DBP, total calorie intake and HOMA-IR.	RCT
Exclusion criteria	People with history of cardiovascular disease or type 1 or 2 diabetes, use of medications that could affect study outcomes, unstable weight for 3 months prior to the beginning of the study (>4-kg weight loss or gain), perimenopause or otherwise irregular menstrual cycle, pregnancy, and currently smoking, etc.	Other therapy.	Other therapy.	Qualitative outcomes such as patient feelings; Inadequate indicators;	Observational study, letters, comments, reviews, and animal experiment.

*ADF, alternate day fasting; RCT, randomized controlled trial; BMI, body mass index; TC, total cholesterol; LDL, low-density lipoprotein; TG, triglycerides; HDL, high-density lipoprotein; FBS, fasting blood sugar; SBP, systolic blood pressure; DBP, diastolic blood pressure; HOMA-IR, homeostasis model assessment-insulin resistance*.

### Quality Assessment

**Figure 1:**
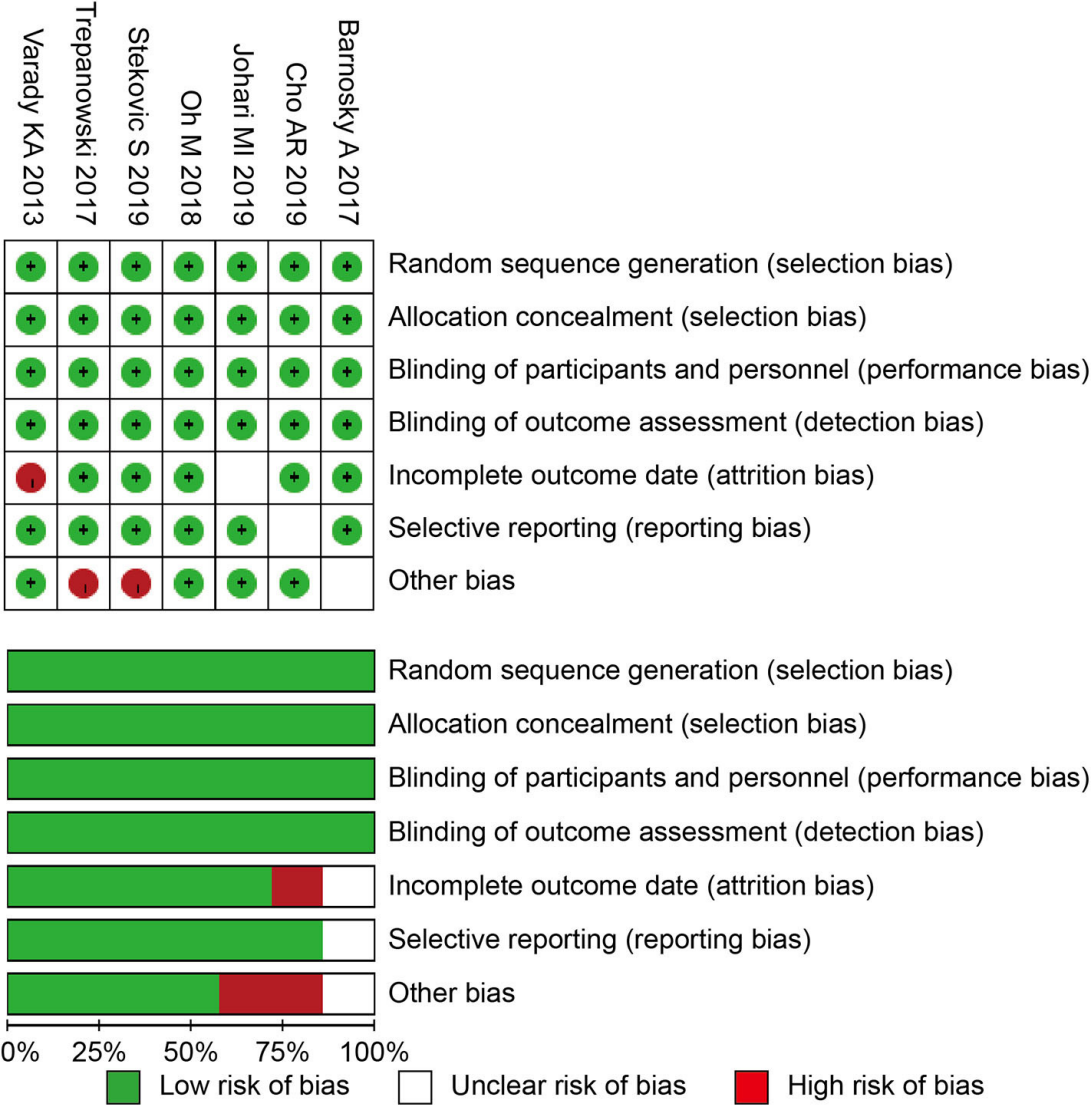
Risk of bias summary and graph. RCT, randomized controlled trials.

The Cochrane risk of bias tool was used to determine the quality of the retrieved RCTs (31). The quality items were selective outcome reporting, blinding, allocation concealment, incomplete outcome data, random sequence generation, and other sources of bias. A graph summarizing the risk of bias was generated based on discussions among the authors, as shown in Figure 1. Then, according to the guidelines published in the Cochrane Handbook for Systematic Reviews of Interventions v.5.3.0, the studies were classified qualitatively (32). All of the authors participated in the quality assessment of all RCTs and agreed with the results. Meanwhile, the differences between each RCT were bridged through discussion among authors. All reviewers independently assessed whether the study was suitable or not according to the criteria.

### Data Extraction

The following information was collected: (a) the general data in the test; (b) name of the first author; (c) time of publication; (d) the design of study and size of the sample (e.g., the interventions between the different groups); (e) the efficacious data that changes in the following parameters, such as weight, body mass index (BMI), total cholesterol (TC), LDL, TG, high-density lipoprotein (HDL), fasting blood sugar (FBS), fat mass, lean mass, systolic blood pressure (SBP), diastolic blood pressure (DBP), total calorie intake, and homeostasis model assessment-insulin resistance (HOMA-IR). Meanwhile, our team cross-checked references and data of each included study to ensure there is no overlapping data.

### Statistical Analysis and Meta-Analysis

The data were carried out using the RevMan version 5.3.0 (Cochrane Collaboration, UK) (32). The changes in the weight, BMI, TC, LDL, TG, HDL, FBS, fat mass, lean mass, SBP, DBP, total calorie intake, and HOMA-IR were analyzed concerning the differences of each RCT between the entry and endpoint. This meta-analysis used mean difference (MD) to evaluate continuous data, and the odds ratio with 95% confidence intervals (CIs) was applied to evaluate dichotomous data. The fixed-effects model was used and considered to be homogeneous if the result was a *p*-value > 0.05. We utilized the I^2^ statistic to analyze inconsistent results, reflecting the proportion of heterogeneity across trials. In this meta-analysis, it is unnecessary to have ethical approval and patient consent because all of the data were acquired from articles that have already been published. A random-effects model would be used in the study when the results showed *p* < 0.05 and *I*^2^ > 50%. Moreover, if the results of the study showed *p* < 0.05, the result was deemed to be statistically significant. Meanwhile, a subgroup analysis was conducted according to the lengths of intervention time (ADF_8W_ and ADF_12W_) in patients (Table 2).

**Table 2:** Subgroup analysis.

	**Weight**	**BMI**	**Total calorie intake**	**TC**	**TG**	**LDL**	**HDL**	**FBS**	**HOMA-IR**	**Fat mass**	**Lean mass**	**SBP**	**DBP**
ADF_8W_	MD −3.13, 95%CI −1.62 to −2.64, *p* < 0.00001	MD −1.20, 95%CI −1.44 to −0.96, *p* < 0.00001	MD −214.68, 95%CI −464.23 to 34.87, *p* = 0.09	MD −12.62, 95%CI −30.10 to 4.85, *p* = 0.16	MD −1.03, 95%CI −4.29 to −2.23, *p* = 0.53	MD −7.46, 95%CI −23.06 to −8.14, *p* = 0.35	MD −0.52, 95%CI −1.88 to −0.84, *p* = 0.45	MD −1.90, 95%CI −5.55 to −1.75, *p* = 0.31	MD −0.38, 95%CI −0.44 to 1.19, *p* = 0.37	MD −2.17, 95%CI −2.96 to −1.38, *p* < 0.00001	MD −0.72, 95%CI −1.43 to −0.01, *p* = 0.05	MD −2.55, 95%CI −4.27 to −0.83, *p* = 0.004	MD −2.54, 95%CI −3.93 to −1.16, *p* = 0.0003
ADF_12W_	MD −5.92, 95%CI −7.36 to −4.47, *p* < 0.00001	NA	MD −80.59, 95%CI −354.81 to 193.66, *p* = 0.56	MD −12.17, 95%CI −21.28 to −3.06, *p* = 0.009	MD −20.16, 95%CI −43.09 to 2.77, *p* = 0.08	MD −6.77, 95%CI −10.55 to −2.99, *p* = 0.0005	MD −0.59, 95%CI −5.67 to −4.49, *p* = 0.82	NA	NA	MD −7.86, 95%CI −14.35 to −1.37, *p* = 0.02	MD −2.35, 95%CI −3.13 to −1.56, *p* < 0.00001	MD −5.97, 95%CI −9.98 to −1.95, *p* = 0.003	MD −4.44, 95%CI −11.10 to 2.21, *p* = 0.19

*ADF, Alternate day fasting; BMI, body mass index; TC, total cholesterol; LDL, low-density lipoprotein; TG, triglycerides; HDL, high-density lipoprotein; FBS, fasting blood sugar; SBP, systolic blood pressure; DBP, diastolic blood pressure; HOMA-IR, homeostasis model assessment-insulin resistance; MD, mean difference; CI, confidence interval; NA, not available*.

## Results

### Characteristics of the Individual Studies

**Figure 2:**
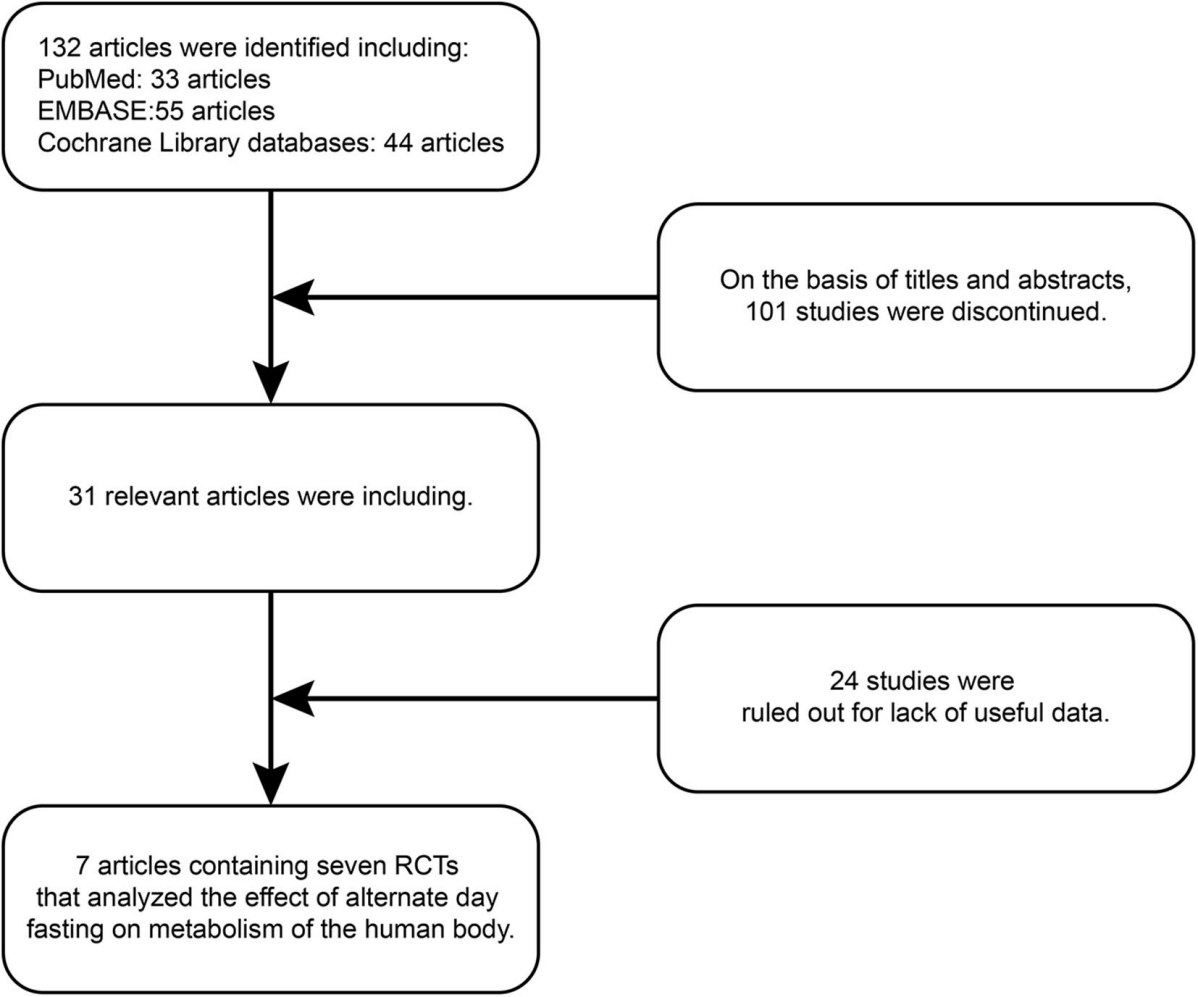
Flowchart of the study selection process. RCT, randomized controlled trials.

One hundred and thirty-two articles were discovered by retrieval in each database. After scrutinizing their abstracts and titles, 101 studies were discontinued. Twenty-four studies were ruled out for a lack of useful data. Finally, seven articles containing seven RCTs (9, 19, 33–37) that analyzed the effect of ADF on the metabolism of the human body were included in our analysis. A detailed flowchart showing the selection process is shown in Figure 2. Table 3 shows the baseline characteristics of the studies.

**Table 3:** Study and patient characteristics.

**Study**	**Country**	**Experimental group**	**Control group**	**Sample size**		**Time of therapy (weeks)**	**Inclusion criteria**	**Exclusion criteria**
				**Experimental**	**Control**			
Varady et al. (19)	Chicago	ADF (4:3)	Permitted to eat ad libitum every	15	15	12	BMI between 20 and 29.9 kg/m2; age between 35 and 65 years; weight stable for 3 months prior to the beginning of the study; non-diabetic; and not taking weight loss, lipid or glucose-lowering medications.	NA
Barnosky et al. (34)	American	ADF (4:3)	Maintain present lifestyle	21	17	24	Men and wome aged 18–65 years with a BMI of 25–39.9 kg/m^2^.	People with history of cardiovascular disease, diabetes mellitus, were taking weight loss medications, were not weight stable for 3 months prior to study initiation.
Trepanowski et al. (9)	American	ADF (4:3)	Maintain present lifestyle	34	31	48	Men and women between 18 and 65 years of age, with a body mass index between 25.0 and 39.9.	People with history of cardiovascular disease or type 1 or 2 diabetes, use of medications that could affect study outcomes, unstable weight for 3 months prior to the beginning of the study.
Minsuk et al. (36)	Korea	ADF (4:3)	Permitted to eat ad libitum every day	13	10	8	Aged 18–64 years; BMI over 23.0 kg/m^2^; no weight variation > 5 kg during the previous 3 months; not diagnosed with type 1 or2 diabetes mellitus; AST or ALT levels <200 mg/dl.	NA
Cho et al. (35)	Canada	ADF (4:3)	Maintain present lifestyle	8	5	8	Age 20-65 years; BMI more than 23.0 kg/ m^2^; stable weight for 3 months prior to the study; no secondary obesity; non-diabetic; AST or ALT levels <200 mg/dL; serum creatinine level <2.0 mg/dL and so on.	NA
Johari et al. (33)	Malaysia	ADF (4:3)	Maintain present lifestyle	33	10	8	ALT > 41 and or AST > 34 IU/L, age that ranged from 18 to 70 years old, BMI between 17.5 and 40 Kg/m2 and no evidence of other forms of liver diseases.	People with significant alcohol consumption, pregnancy, and involvement in an active weight loss program or taking weight loss medications.
Stekovic et al. (37)	France	ADF	Maintain present lifestyle	28	29	4	Age between 35 and 65 years; BMI between 22.0 and 30.0 kg/m2, both inclusive; Stable weight for 3 months prior to the study; Stable weight for 3 months immediately prior to the study.	History of metabolic disorder, history of cardiovascular disease, acute or chronic inflammatory disorder, known malignancy.

### Quality of the Individual Studies

**Figure 3:**
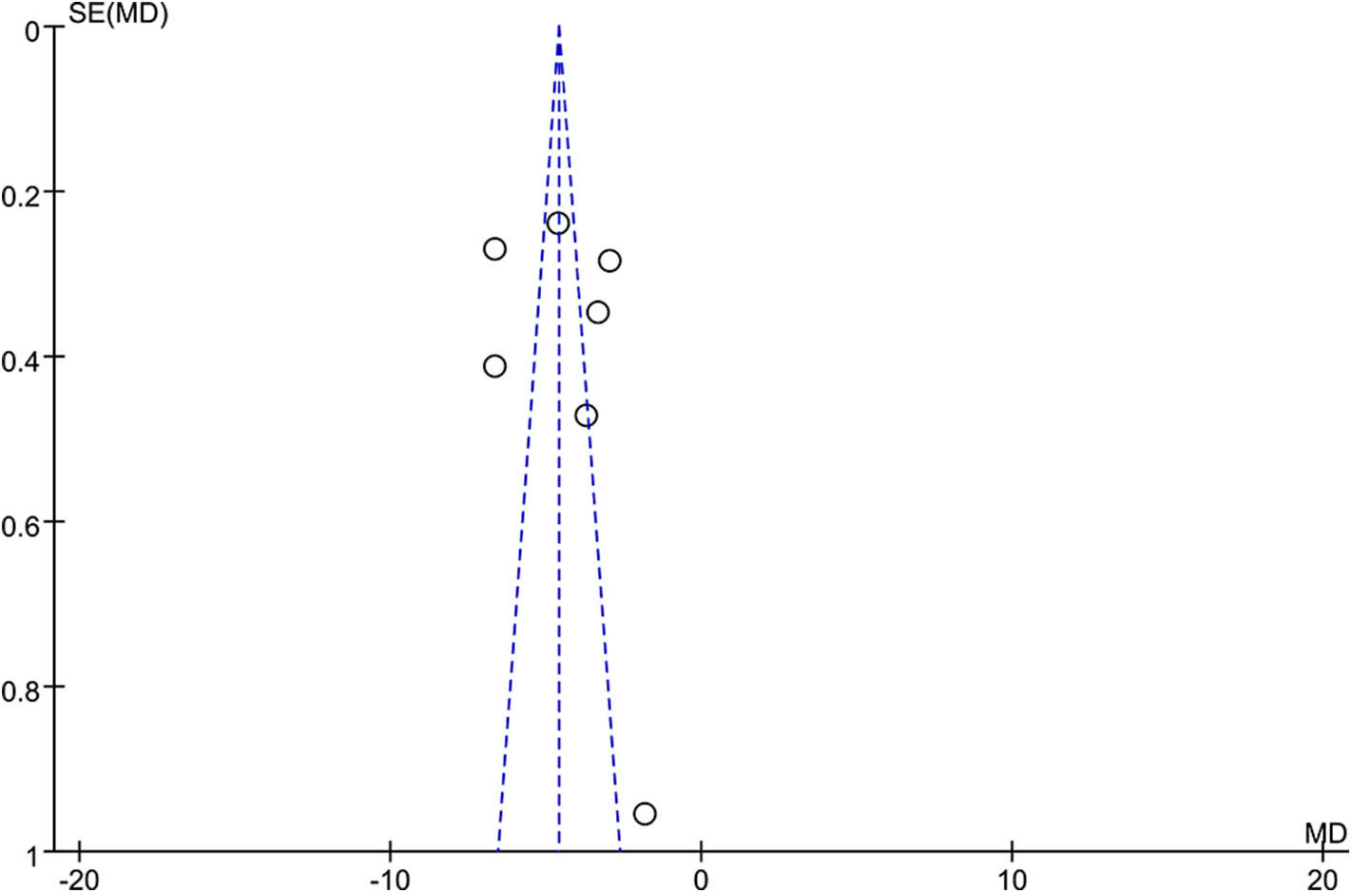
Funnel plot of the studies included in our meta-analysis. MD, mean difference; SE, standard error.

All of the seven studies included in the meta-analysis were RCT. Figure 1 presents a graphical summary of the risk bias. Besides, all of the studies described the randomization process. All articles had an appropriate number of participants to analyze. The funnel plot displayed the conclusion of a qualitative estimation of publication bias (Figure 3).

### Efficacy

#### Weight

**Figure 4:**
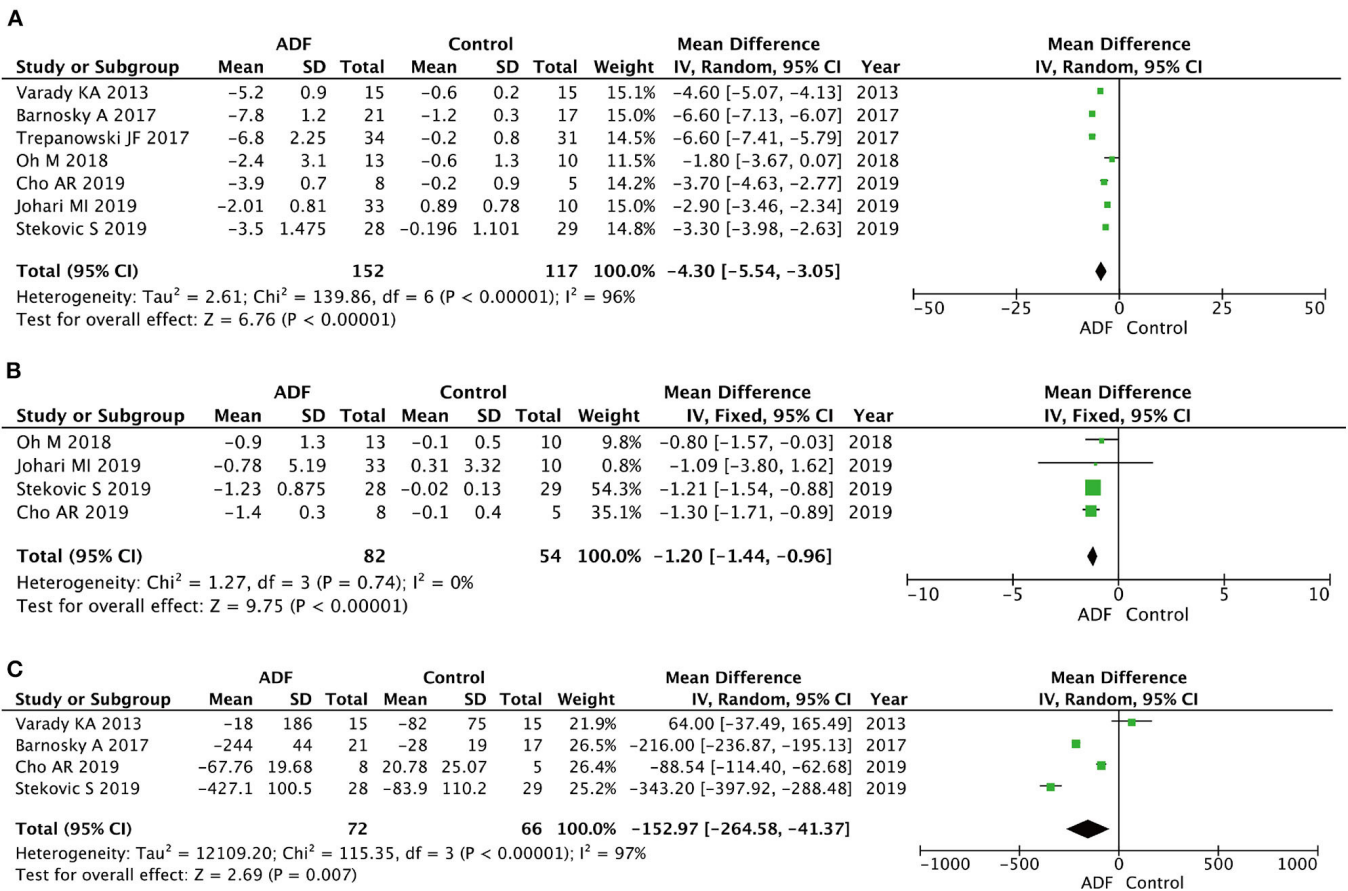
Forest plots showing changes between two groups in **(A)** weight, **(B)** body mass index (BMI), **(C)** total calorie intake; SD, standard deviation; IV, inverse variance; CI, confidence interval; df, degrees of freedom.

Seven RCTs involving 269 participants contained meaningful data on weight (152 in the ADF group and 117 in the control group). A random-effects model was adopted to evaluate changes between the two groups, showing an MD of −4.30, 95% CI: −5.54 to −3.05, *P* < 0.00001. It proved that compared with the control group (Figure 4), the ADF group showed statistically significant reductions in weight.

#### Body Mass Index

Four RCTs involving 136 participants contained meaningful data on BMI (82 in the ADF group and 54 in the control group). A fixed-effects model was adopted to evaluate changes between the two groups, showing an MD of −1.20, 95% CI: −1.44 to −0.96, *P* < 0.00001. The result proved that the ADF group showed statistical differences in BMI compared with the control group (Figure 4).

#### Total Calorie Intake

Four RCTs involving 138 participants contained meaningful data on total calorie intake (72 in the ADF group and 66 in the control group). A random-effects model was adopted to evaluate changes between the two groups, showing an MD of −152.97, 95% CI: −264.58 to −41.37, *p* = 0.007. It demonstrated that the ADF group showed statistically significant reductions in total calorie intake compared with the control group (Figure 4).

#### Total Cholesterol

**Figure 5:**
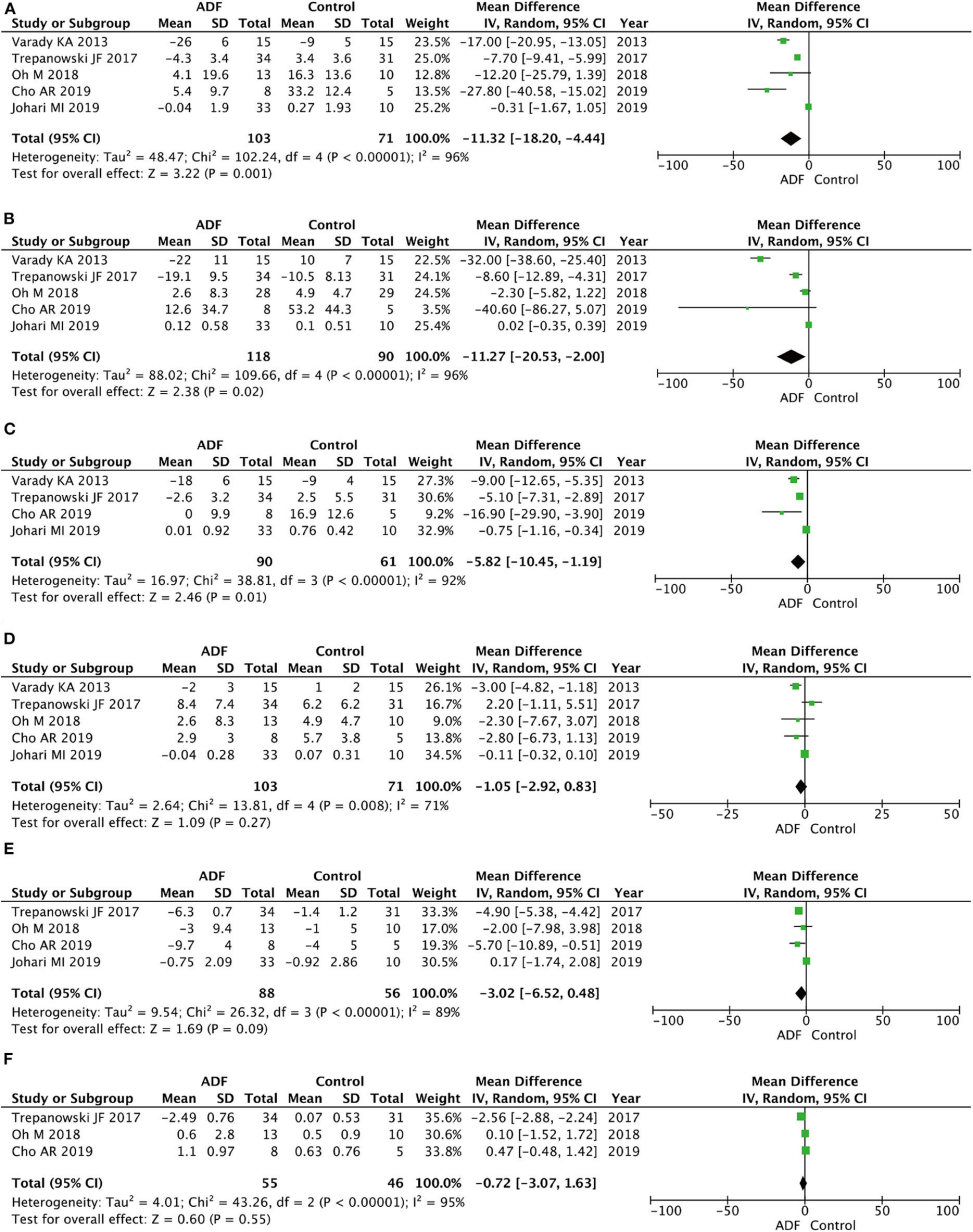
Forest plots showing changes between two groups in **(A)** total cholesterol (TC), **(B)** triglycerides (TG), **(C)** low-density lipoprotein (LDL), **(D)** high-density lipoprotein (HDL), **(E)** fasting blood sugar (FBS), **(F)** homeostasis model assessment-insulin resistance (HOMA-IR); SD, standard deviation; IV, inverse variance; CI, confidence interval; df, degrees of freedom.

Five RCTs involving 174 participants contained meaningful data on TC (103 in the ADF group and 71 in the control group). A random-effects model was chosen to evaluate changes between the two groups, showing an MD of −11.32, 95% CI: −18.20 to 4.44, *P* = 0.001. We found significant differences between the ADF group and the control group in the TC (Figure 5).

#### Triglycerides

Five RCTs involving 208 participants contained meaningful data on TG (118 in the ADF group and 90 in the control group). A random-effects model was chosen to evaluate changes between the two groups, which showed an MD of −11.27, 95% CI: −20.53 to −2.00, *P* = 0.02. The result proved that the ADF group showed significant differences in TG compared with the control group (Figure 5).

#### Low-Density Lipoprotein

Four RCTs involving 151 participants contained meaningful data on LDL (90 in the ADF group and 61 in the control group). A random-effects model was adopted to evaluate changes between the two groups, showing an MD of −5.82, 95% CI: −10.45 to −1.19, *P* = 0.01. The result proved that the ADF group showed significant differences in LDL compared with the control group (Figure 5).

#### High-Density Lipoprotein

Five RCTs involving 174 participants contained meaningful data on HDL (103 in the ADF group and 71 in the control group). A random-effects model was chosen to evaluate changes between the two groups, which showed an MD of −1.05, 95% CI: −2.92 to 0.83, *P* = 0.27. The result showed that it was no statistical difference in terms of HDL between the two groups (Figure 5).

#### Fasting Blood Sugar

Four RCTs involving 144 participants contained meaningful data on FBS (88 in the ADF group and 56 in the control group). A random-effects model was chosen to evaluate changes between the two groups, showing an MD of −3.02, 95% CI: −6.52 to 0.28, *P* = 0.09. The model showed no marked differences between the ADF group and the control group in the change of FBS (Figure 5).

#### Homeostasis Model Assessment—Insulin Resistance

Three RCTs involving 101 participants contained meaningful data on HOMA-IR (55 in the ADF group and 46 in the control group). A random-effects model was used to evaluate changes between the two groups, showing an MD of −0.72, 95% CI: −3.07 to 1.63, *p* = 0.55. Compared with the control group, the ADF group showed no meaningful difference in HOMA-IR (Figure 5).

#### Fat Mass and Lean Mass

**Figure 6:**
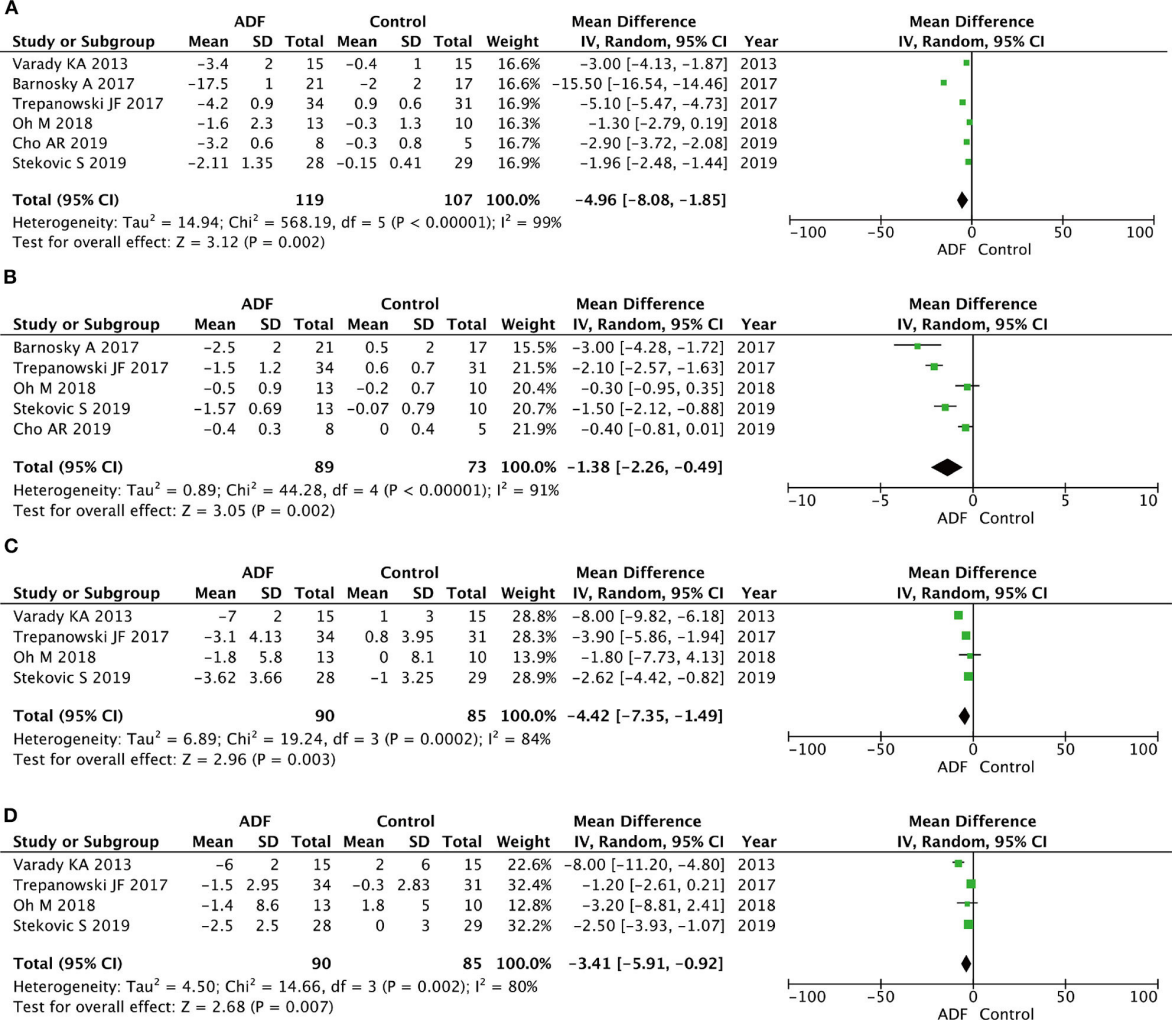
Forest plots showing changes between two groups in **(A)** fat mass, **(B)** lean mass, **(C)** systolic blood pressure (SBP), **(D)** diastolic blood pressure (DBP); total calorie intake; SD, standard deviation; IV, inverse variance; CI, confidence interval; df, degrees of freedom.

Six RCTs involving 226 participants contained meaningful data on fat mass (119 in the ADF group and 107 in the control group). In terms of lean mass, five RCTs had an appropriate sample size of 172 patients (89 in the ADF group and 73 in the control group). A random-effects model showed statistical differences between the ADF group and the control group in the change of fat mass (MD −4.96, 95%CI −8.08 to 1.85, *p* = 0.002) and lean mass (MD −1.38, 95%CI −2.26 to −0.49, *p* = 0.002) (Figure 6).

#### Systolic Blood Pressure and Diastolic Blood Pressure

Four RCTs involving 175 participants contained meaningful data on SBP and DBP (90 in the ADF group and 85 in the control group). A random-effects model was chosen to estimate changes between the two groups. The result demonstrated that the ADF group showed statistical differences in SBP (MD −4.42, 95%CI −7.35 to −1.49, *p* = 0.003) and DBP (MD −3.41, 95%CI −5.91 to −0.92, *p* = 0.007) compared with the control group (Figure 6).

### Subgroup Analysis

In subgroup analyses, both ADF_8W_ and ADF_12W_ improved anthropometric outcomes, such as body weight (ADF_8W_: *p* < 0.00001; ADF_12W_: *p* < 0.00001), fat mass (ADF_8W_: *p* < 0.00001; ADF_12W_: *p* = 0.02), and SBP (ADF_8W_: *p* = 0.004; ADF_12W_: *p* = 0.003). Meanwhile, comparing ADF_8W_ and ADF_12W_ with control, ADF_8W_ performed slightly better than ADF_12W_ for total calorie intake (ADF_8W_: *p* = 0.09; ADF_12W_: *p* = 0.56) and DBP (ADF_8W_: *p* = 0.0003; ADF_12W_: *p* = 0.19), ADF_12W_ performed slightly better for TC (ADF_8W_: *p* = 0.16; ADF_12W_: *p* = 0.009), lean mass (ADF_8W_: *p* = 0.05; ADF_12W_: *p* < 0.00001), and LDL (ADF_8W_: *p* = 0.35; ADF_12W_: *p* = 0.0005). No subgroup differences were observed for HDL (ADF_8W_: *p* = 0.45; ADF_12W_: *p* = 0.82) and TG (ADF_8W_: *p* = 0.53; ADF_12W_: *p* = 0.08) (Table 2).

## Discussion

Recently, the most commonly used diet strategy for weight loss is calorie restriction. In many parts of the world, ADF is an age-old way that includes many forms. For example, Ramadan is a form of ADF and abided by Muslims all around the world (38). If someone wants to lose weight, it is generally recommended to restrict diets and exercises in clinical practice guidelines (39, 40). The traditional methods of losing weight (such as the daily calorie restriction), although the effect is affirmative, the control and compliance are poor (41). Based on this foundation, intermittent fasting regimens, especially ADF protocols, are being proposed in many pieces of literature (9, 19, 33–37). The important unsolved issue is whether the effects of ADF can play a role for people who want to lose weight.

This quantitative meta-analysis summarized the evidence from RCTs. We performed this meta-analysis from seven studies, including 269 participants, to evaluate the effect of ADF on weight loss for at least 1 month. In this meta-analysis, compared with the control group, the ADF group showed statistically significant reductions in weight (*p* < 0.00001) and BMI (*p* < 0.00001). In this case, we selected some meaningful indexes of many diverse indicators to illustrate this difference between the two groups. Here, the ADF group showed significant differences in terms of TC (*p* = 0.001), LDL (*p* = 0.01), TG (*p* = 0.02), fat mass (*p* = 0.002), lean mass (*p* = 0.002), SBP (*p* = 0.003), DBP (*p* = 0.007), and total calorie intake (*p* = 0.007) compared with the control group. At the same time, the analysis demonstrated that the ADF group had a similar effect compared with the control group in aspects of HDL (*P* = 0.27) and FBS (*P* = 0.09). Based on our results, ADF was the positive influential method on the physiology, body composition, and parameters for obesity or a normal human.

For the first time, this meta-analysis suggested that ADF is a potentially superior alternative to daily calorie restriction in normal-weight and overweight subjects. Just the way we assumed, the ADF strategy was effectively reduced body-related biomarkers, such as weight, BMI, and so on. Compared with the complete calorie restriction method, ADF had a lower capacity for weight management. In animal experiments, weight loss can redistribute fat in the ADF group without losing lean mass (42). New research showed that exercise plus ADF would experience the largest reductions in cardiometabolic risk factors, with the least decrease in lean mass compared with ADF alone (36). Also, it could be suggested as an alternative option for daily calorie restriction (CR) in treating nonalcoholic fatty liver disease (33). On the one hand, the decrease of liver enzymes might be explained by an improvement in visceral fat or steatosis of the liver in animal and human experiments. On the other hand, the liver enzymes have an increased anomaly in “fast days” and reduced in “feast days,” so this is an irreversible process (43). The phenomenon showed that ADF could promote hepatocyte restorative process when transient autophagy occurred to liver cells (44). However, the exact molecular mechanisms that underlie fasting and liver autophagy need to be further studied and established (45).

The research suggested that ADF had effects on cardiovascular improvements. As is known to all, alterations in cholesterol metabolism were known to be powerful predictors of developing cardiovascular events, even in the early stages of atherosclerosis (46). For instance, abnormal cholesterol metabolism, including low intestinal cholesterol absorption and elevated cholesterol biosynthesis, played an important role in metabolic syndrome, obesity, and diabetes (47). This diet strategy may also have cardioprotective effects in participants by reducing triacylglycerol and increasing LDL particle size and adiponectin concentration. To our knowledge, the Mediterranean and certain low carbohydrate diets help maintain a healthy weight and reduce the risks of coronary heart disease. If ADF were combined with a Mediterranean diet or a low-carbohydrate diet, it would be meaningful to observe how it affects weight loss and cardiovascular outcomes in future studies (48, 49). To improve lipid, the combination of diet and exercise is more effective than diet or exercise alone (50). This diet strategy may also have cardioprotective effects in participants by reducing triacylglycerol and increasing LDL particle size and adiponectin concentration. Previous research has already demonstrated that glucose and insulin have been associated with obesity. Thus, it is important to manage and control glucose levels and insulin resistance (51). At the same time, the significant decrease in fasting insulin may potentially be attributed in part to the decline in body weight and the reduction in total body fat (52). We found a significant reduction in lean mass in the ADF group. This serves as a caution for patient populations at risk for sarcopenia because ADF could exacerbate muscle loss. Some studies have shown that adequate/excessive protein consumption during weight loss can mitigate losses in lean mass (53).

Some studies indicated that ADF is the most beneficial diet strategy for lowering fasting insulin, glucose, and HOMA-IR. However, there was no significant difference in insulin resistance between the two groups. It suggested that ADF plus exercise might reduce insulin resistance, which needs to be further elucidated (36). Meanwhile, ADF did not result in a decline in bone mineral density or white blood cell count. It might even have a trend to increase bone mineral density values for a long period (54–56). It was worth mentioning that no effect was found in some studies: (a) the weight of participants lost <10%; (57, 58) (b) the sample size of RCTs that we selected was weak; (c) the duration of the maintenance phase was absent; (d) others, such as the time for blood sampling and so on. In a recent study we founded, ADF likely makes little differences compared with continuous energy restriction, but ADF probably slightly reduces body weight and fat mass. In additional analyses, no important differences were detected when comparing different types of ADF (4:3 vs. 5:2; consecutive vs. nonconsecutive days) (59).

Randomized control trials suggested that ADF is effective for weight loss, weight maintenance, and improving certain metabolic disease risk factors such as LDL cholesterol, blood pressure, and fasting insulin after 6 months (60). However, all studies were limited by a lower number of included RCTs and were often limited by also including short-term trials (<12 weeks) (61, 62).

ADF had some physiological benefits with similar daily calorie restriction (63). Recently, true ADF that had a limited capacity intake completely on a fast day was demonstrated and reduced fat cell size by 35–55% in both visceral and subcutaneous adipose tissue depots after 4 weeks in mice (64). ADF might lead to fat redistribution from visceral to subcutaneous depots in female mice (42). Evidence suggested that plasma adiponectin was inversely proportional to visceral fat accumulation (65, 66). Thus, the redistribution in body fat by ADF may be linked to increases in plasma adiponectin observed. All in all, ADF may improve body fat distribution and circulating adiponectin; conversely, the diet strategy may take precautions against the development of obesity-related diseases whether these effects can be reproduced in clinical trials.

Several adverse events were reported in the study. Its incidences were significantly low and slight. A small number of participants experienced mild headaches or light-headedness in the early days of the trial. Others reported constipation during weeks 1 and 2 of the trial (27). This phenomenon may or may not be linked with dietary therapy. The participants were proposed to be consuming more fruits and vegetables on feed days. Those clinical manifestations would gradually disappear in the course of the experiment.

This meta-analysis included seven RCTs and concentrating on the efficacy of ADF in participants. Compared with previous studies, our study had some advantages; the data were derived from randomized, double-blind, controlled trials. However, this study also has some limitations, which reflect the common limitations of other systematic reviews and meta-analyses. First of all, this article did not include numerous RCTs such as unpublished studies, which limit evidence to affect study quality. Second, RCTs had low methodological rigor and short intervention; more appropriate high-quality trials are needed to improve the accuracy of results.

## Conclusions

In summary, this meta-analysis suggests that ADF is a viable diet strategy for weight loss, and it has a substantial improvement in risk indicators for diseases in obese or normal people. Therefore, adults, whether healthy or not, should perform ADF with recommendations of clinical physicians to prevent adverse effects.

## Data Availability Statement

The original contributions presented in the study are included in the article/supplementary materials, further inquiries can be directed to the corresponding author/s.

## Author Contributions

YL: literature search. ZG: study design and data collection. TC: data interpretation. JW and YZ: writing. All authors contributed to the article and approved the submitted version.

## Conflict of Interest

The authors declare that the research was conducted in the absence of any commercial or financial relationships that could be construed as a potential conflict of interest.

## Abbreviations

ADF: alternate day fasting
RCTs: randomized controlled trials
TC: total cholesterol
BMI: body mass index
LDL: low-density lipoprotein
LDL: low-density lipoprotein
TG: triglycerides
SBP: systolic blood pressure
DBP: diastolic blood pressure
HDL: high-density lipoprotein
HOMA-IR: homeostasis model assessment-insulin resistance
FBS: fasting blood sugar
MD: mean difference
OR: odds ratio
CI: confidence intervals
CR: calorie restriction.

## References

[1] Wilson PW D'Agostino RB Sullivan L Parise H Kannel WB. Overweight and obesity as determinants of cardiovascular risk: the Framingham experience. Arch Intern Med. (2002) 162:1867–72. 10.1001/archinte.162.16.1867 12196085

[2] Popkin BM Gordon-Larsen P. The nutrition transition: worldwide obesity dynamics and their determinants. Int J Obes Relat Metab Dis. (2004) 28:S2. 10.1038/sj.ijo.0802804 15543214

[3] Kroemer G López-Otín C Madeo F De Cabo R. Carbotoxicity—noxious effects of carbohydrates. Cell. (2018) 175:605–614. 10.1016/j.cell.2018.07.044 30340032 PMC6265656

[4] Curioni CC LourenO PM. Long-term weight loss after diet and exercise: a systematic review. Int J Obes. (2005) 29:1168–74. 10.1038/sj.ijo.0803015 15925949

[5] Villareal DT Chode S Parimi N Sinacore DR Hilton T Armamento-Villareal R Weight loss, exercise, or both and physical function in obese older adults. N Engl J Med. (2011) 364:1218–29. 10.1056/NEJMoa1008234 21449785 PMC3114602

[6] Miller WC Koceja DM Hamilton EJ A meta-analysis of the past 25 years of weight loss research using diet, exercise or diet plus exercise intervention. Int J Obes Relat Metab Disord. (1997) 21:941–7. 10.1038/sj.ijo.0800499 9347414

[7] Wu T Gao X Chen M Dam RMV. Long-term effectiveness of diet-plus-exercise interventions vs. diet-only interventions for weight loss: a meta-analysis. Obes Rev. (2009) 10:313–23. 10.1111/j.1467-789X.2008.00547.x 19175510

[8] Wood PD Stefanick ML Williams PT Haskell WL The effects on plasma lipoproteins of a prudent weight-reducing diet, with or without exercise, in overweight men and women. J Cardiopulm Rehabil. (1991) 325:461–6. 10.1056/NEJM199108153250703 1852180

[9] Trepanowski JF Kroeger CM Barnosky A Klempel MC Bhutani S Hoddy KK. Effect of alternate-day fasting on weight loss, weight maintenance, and cardioprotection among metabolically healthy obese adults. JAMA Intern Med. (2017) 177:930–8. 10.1001/jamainternmed.2017.0936 28459931 PMC5680777

[10] Hansen D Dendale P Berger J van Loon LJC. The effects of exercise training on fat-mass loss in obese patients during energy intake restriction. Sports Med. (2007) 37:31–46. 10.2165/00007256-200737010-00003 17190534

[11] Ross R Rissanen J Pedwell H Clifford J Shragge P. Influence of diet and exercise on skeletal muscle, and visceral adipose tissue in men. J Appl Physiol. (1996) 81:2445–55. 10.1152/jappl.1996.81.6.2445 9018491

[12] Fock KM Khoo J. Diet and exercise in management of obesity and overweight. J Gastroenterol Hepatol. (2013) 28:59–63. 10.1111/jgh.12407 24251706

[13] Jensen MD Ryan DH Apovian CM Ard JD Comuzzie AG Donato KA. aha/acc/tos guideline for the management of overweight and obesity in adults. J Am Coll Cardiol. (2014) 63:2985–3023. 10.1016/j.jacc.2013.11.004 24239920

[14] Moreira EAM Most M Howard J Ravussin E. Dietary adherence to long-term controlled feeding in a calorie-restriction study in overweight men and women. Nutr Clin Pract. (2011) 26:309–15. 10.1177/0884533611405992 21586416 PMC4830337

[15] Moroshko I Brennan L O"Brien P. Predictors of dropout in weight loss interventions: a systematic review of the literature. Obes Rev. (2011) 12:912–34. 10.1111/j.1467-789X.2011.00915.x 21815990

[16] Antoni R Johnston KL Collins AL Robertson MD. Intermittent v. continuous energy restriction: differential effects on postprandial glucose and lipid metabolism following matched weight loss in overweight/obese participants. Br J Nutr. (2018) 119:507–16. 10.1017/S0007114517003890 29508693

[17] Mattison JA Colman RJ Beasley TM Allison DB Kemnitz JW Roth GS. Caloric restriction improves health and survival of rhesus monkeys. Nat Commun. (2017) 8:14063. 10.1038/ncomms14063 28094793 PMC5247583

[18] Redman LM Heilbronn LK Martin CK de Jonge L Williamson DA Delany JP. Metabolic and behavioral compensations in response to caloric restriction: implicationsfor the maintenance of weight loss. PLoS ONE. (2009) 4:e4377. 10.1371/journal.pone.0004377 19198647 PMC2634841

[19] Varady KA Bhutani S Klempel MC Kroeger CM Trepanowski JF. Alternate day fasting for weight loss in normal weight and overweight subjects: a randomized controlled trial. Nutr J. (2013) 12:1–8. 10.1186/1475-2891-12-146 24215592 PMC3833266

[20] Varady KA Bhutani S Church EC Klempel MC. Short-term modified alternate-day fasting: a novel dietary strategy for weight loss and cardioprotection in obese adults. Am J Clin Nutr. (2000) 5:1138–43. 10.3945/ajcn.2009.28380 19793855

[21] Klempel MC Kroeger CM Bhutani S Trepanowski JF Varady KA. Intermittent fasting combined with calorie restriction is effective for weight loss and cardio-protection in obese women. Nutr J. (2012) 11:98. 10.1186/1475-2891-11-98 23171320 PMC3511220

[22] Varady KA Hellerstein MK. Alternate-day fasting and chronic disease prevention: a review of human and animal trials. Am J Clin Nutr. (2007) 86:7–13. 10.1093/ajcn/86.1.7 17616757

[23] Luigi F. Aging, adiposity, and calorie restriction. JAMA. (2007) 297:986. 10.1001/jama.297.9.986 17341713

[24] Tinsley GM La BPM. Effects of intermittent fasting on body composition and clinical health markers in humans. Nutr Rev. 73:661–74. 10.1093/nutrit/nuv041 26374764

[25] Varady KA Bhutani S Church EC Klempel MC. Short-term modified alternate-day fasting: a novel dietary strategy for weight loss and cardioprotection in obese adults. Am J Clin Nutr. (2000) 5. 19793855 10.3945/ajcn.2009.28380

[26] Hoddy KK Kroeger CM Trepanowski JF Barnosky A Bhutani S Varady KA. Meal timing during alternate day fasting: impact on body weight and cardiovascular disease risk in obese adults. Obesity. (2014) 22:2524–31. 10.1002/oby.20909 25251676

[27] Ganesan K Habboush Y Sultan S. Intermittent fasting: the choice for a healthier lifestyle. Cureus. (2018) 10:e2947. 10.7759/cureus.2947 30202677 PMC6128599

[28] Mosley M Spencer M The Fastdiet: Lose Weight, Stay Healthy, and Live Longer With the Simple Secret of Intermittent Fasting. Atria Books. Britain (2013).

[29] The 5:2 Fast Diet For Beginners. Berkeley, CA: Rockridge Press (2013).

[30] Moher D Corrigendum to: preferred reporting items for systematic reviews and meta-analyses: the PRISMA statement. Int J Surg. (2010) 8:336–41. 10.1016/j.ijsu.2010.02.007 20171303

[31] Vader JP. Randomised controlled trials: a user's guide. BMJ. (1998) 317:1258. 10.1136/bmj.317.7167.1258 9794885 PMC1114182

[32] DerSimonian R Laird N. Meta-analysis in clinical trials. Control Clin Trials. (1986) 7:177–88. 10.1016/0197-2456(86)90046-2 3802833

[33] Johari MI Yusoff K Haron J Nadarajan C Ibrahim KN Wong MS. A randomised controlled trial on the effectiveness and adherence of modified alternate-day calorie restriction in improving activity of non-alcoholic fatty liver disease. Sci Rep. (2019) 9:11232. 10.1038/s41598-019-47763-8 31375753 PMC6677794

[34] Barnosky A Kroeger CM Trepanowski JF Klempel MC Bhutani. Effect of alternate day fasting on markers of bone metabolism: an exploratory analysis of a 6-month randomized controlled trial. Nutr Healthy Aging. (2017) 4:255–263. 10.3233/NHA-170031 29276795 PMC5734119

[35] Cho AR Moon JY Kim S An KY Oh M Jeon JY Effects of alternate day fasting and exercise on cholesterol metabolism in overweight or obese adults: a pilot randomized controlled trial. Metab Clin Exp. (2019) 93:52–60. 10.1016/j.metabol.2019.01.002 30615947

[36] Minsuk O Sue K Ki-Yong A Jihee M In YH Junga L. Effects of alternate day calorie restriction and exercise on cardio-metabolic risk factors in overweight and obese adults: an exploratory randomized controlled study. BMC Public Health. (2018) 18:1124. 10.1186/s12889-018-6009-1 30219052 PMC6139127

[37] Stekovic S Hofer SJ Tripolt N Aon MA Royer P Pein L. Alternate day fasting improves physiological and molecular markers of aging in healthy, non-obese humans. Cell Metab. (2020) 31:878–81. 10.1016/j.cmet.2020.02.011 32268118

[38] Mohsen M Peyman R Owais C Mohsen N. The effect of Ramadan fasting on cardiovascular risk factors and anthropometrics parameters: a systematic review. Pak J Med Sci. (2015) 31:1250–5. 10.12669/pjms.315.7649 26649024 PMC4641293

[39] European Association for the Study of the Liver (EASL) European Association for the Study of Diabetes (EASD) European Association for the Study of Obesity (EASO) EASL-EASD-EASO clinical practice guidelines for the management of non-alcoholic fatty liver disease. J Hepatol. (2016) 64:1388–402. 10.1016/j.jhep.2015.11.004 27062661

[40] Chitturi S Wong WS Chan WK Wong LH Wong KH. The asia-pacific working party on nonalcoholic fatty liver disease guidelines 2017 part 2: management and special groups. J Gastroenterol Hepatol. (2017) 33:86–98. 10.1111/jgh.13856 28692197

[41] Jeffery RW Drewnowski A Epstein LH Stunkard AJ Hill DR. Long-term maintenance of weight loss: current status. Health Psychol. (2000) 19 (1 Suppl.):5–16. 10.1037/0278-6133.19.Suppl1.5 10709944

[42] Varady KA Allister CA Roohk DJ Hellerstein MK. Improvements in body fat distribution and circulating adiponectin by alternate-day fasting versus calorie restriction. J Nutr Biochem. (2010) 21:188–95. 10.1016/j.jnutbio.2008.11.001 19195863

[43] Brandhorst S Choi IY Wei M Cheng CW Sedrakyan S Navarrete G. A periodic diet that mimics fasting promotes multi-system regeneration, enhanced cognitive performance, and healthspan. Cell Metab. (2015) 22:86–99. 10.1016/j.cmet.2015.05.012 26094889 PMC4509734

[44] Allaire M Rautou PE Codogno P Lotersztajn. Autophagy in liver diseases: time for translation? J Hepatol. (2019) 70:985–98. 10.1016/j.jhep.2019.01.026 30711404

[45] Saito T. Autophagy regulates lipid metabolism through selective turnover of ncor1. Nat Commun. (2019) 10:1567. 10.1038/s41467-019-08829-3 30952864 PMC6450892

[46] Son HH Moon JY Seo HS Kim HH Chung BC Choi MH. High-temperature GC-MS-based serum cholesterol signatures may reveal sex differences in vasospastic angina. J Lipid Res. (2014) 55:155–62. 10.1194/jlr.D040790 24220886 PMC3927468

[47] Wilund KR Feeney LA Tomayko EJ Weiss EP Hagberg JM. Effects of endurance exercise training on markers of cholesterol absorption and synthesis. Physiol Res. (2009) 58:545–52. 10.1109/TMAG.2004.838741 18656998

[48] Kris-Etherton P Eckel RH Howard BV St Jeor S Bazzarre TL Nutrition Committee Population Science Committee and Clinical Science Committee of the American Heart Association. AHA science advisory: lyon diet heart study. Benefits of a mediterranean-style, National Cholesterol Education Program/American Heart Association Step I dietary pattern on cardiovascular disease. Circulation. (2001) 103:1823–5. 10.1161/01.CIR.103.13.1823 11282918

[49] Schwingshackl L Hoffmann G. Low-carbohydrate diets and cardiovascular risk factors. Obes Rev. (2013) 14:183–4. 10.1111/j.1467-789X.2012.01060.x 23294905

[50] Kelley GA Kelley KS Roberts S Haskell W Comparison of aerobic exercise, diet or both on lipids and lipoproteins in adults: a meta-analysis of randomized controlled trials. Clin Nutr. (2012) 31:156–67. 10.1016/j.clnu.2011.11.011 22154987 PMC3311746

[51] Abbasi F Brown BW Lamendola C Mclaughlin T Reaven GM. Relationship between obesity, insulin resistance, and coronary heart disease risk. J Am Coll Cardiol. (2002) 40:937–43. 10.1016/S0735-1097(02)02051-X 12225719

[52] Harris L Hamilton S Azevedo LB Olajide J Ells L. Intermittent fasting interventions for treatment of overweight and obesity in adults: a systematic review and meta-analysis. JBI Database Syst Rev Implem Rep. (2018) 16:507–47. 10.11124/JBISRIR-2016-003248 29419624

[53] Lowe DA Wu N Rohdin-Bibby L. Effects of time-restricted eating on weight loss and other metabolic parameters in women and men with overweight and obesity: the TREAT randomized clinical trial. JAMA Intern Med. (2020) 180:1–9. 10.1001/jamainternmed.2020.4153 32986097 PMC7522780

[54] Schafer AL. Decline in bone mass during weight loss: a cause for concern? J Bone Miner Res. (2016) 31:36–9. 10.1002/jbmr.2754 26595270

[55] Villareal DT Fontana L Das SK Redman L Smith SR Saltzman E Effect of two-year caloric restriction on bone metabolism and bone mineral density in non-obese younger adults: a randomized clinical trial. J Bone Miner Res. (2016) 31:40–51. 10.1002/jbmr.2701 26332798 PMC4834845

[56] Wiklund P Toss F Weinehall L Hallmans G Franks PW NordstrM A Abdominal and gynoid fat mass are associated with cardiovascular risk factors in men and women. J Clin Endocrinol Metab. (2008) 93:4360–6. 10.1210/jc.2008-0804 18728169

[57] Dattilo AM Kris-Etherton PM. Effects of weight reduction on blood lipids and lipoproteins: a meta-analysis. Am J Clin Nutr. (1992) 56:320–8. 10.1093/ajcn/56.2.320 1386186

[58] Jae SY Fernhall B Heffernan KS Jeong M Chun EM Sung J. Effects of lifestyle modifications on c-reactive protein: contribution of weight loss and improved aerobic capacity. Metab Clin Exp. (2006) 55:825–31. 10.1016/j.metabol.2006.02.010 16713444

[59] Schwingshackl L Zähringer J Nitschke K Torbahn G Lohner S Kühn T. Impact of intermittent energy restriction on anthropometric outcomes and intermediate disease markers in patients with overweight and obesity: systematic review and meta-analyses. Crit Rev Food Sci Nutr. (2020) 1–12. 10.1080/10408398.2020.1757616 32363896

[60] Kalam F Gabel K Cienfuegos S Wiseman E Ezpeleta M Steward M. Alternate day fasting combined with a low-carbohydrate diet for weight loss, weight maintenance, and metabolic disease risk reduction. Obesity science practice. (2019) 5:531–9. 10.1002/osp4.367 31890243 PMC6934424

[61] Cho Y Hong N Kim KW Cho SJ Lee M Lee YH. The effectiveness of intermittent fasting to reduce body mass index and glucose metabolism: a systematic review and meta-analysis. J Clin Med. (2019) 8:1645. 10.3390/jcm8101645 31601019 PMC6832593

[62] Cioffi I Evangelista A Ponzo V Ciccone G Soldati L Santarpia L. Intermittent versus continuous energy restriction on weight loss and cardiometabolic outcomes: a systematic review and meta-analysis of randomized controlled trials. J Transl Med. (2018) 16:371. 10.1186/s12967-018-1748-4 30583725 PMC6304782

[63] Varady KA Roohk DJ McEvoyHein BK Gaylinn BD Thorner MO Hellerstein MK. Modified alternate-day fasting regimens reduce cell proliferation rates to a similar extent as daily calorie restriction in mice. FASEB J. (2008) 22:2090. 10.1096/fj.07-098178 18184721 PMC2975447

[64] Varady KA Roohk DJ Loe YC Mcevoy-Hein BK Hellerstein MK. Effects of modified alternate-day fasting regimens on adipocyte size, triglyceride metabolism, and plasma adiponectin levels in mice. J Lipid Res. (2007) 48:2212–9. 10.1194/jlr.M700223-JLR200 17607017

[65] Asayama K Hayashibe H Dobashi K. Decrease in serum adiponectin level due to obesity and visceral fat accumulation in children. Obes Res. (2003). 11:1072–9. 10.1038/oby.2003.147 12972677

[66] Rodríguez A Catalán V Gómez-Ambrosi J Fruhbeck G. Visceral and subcutaneous adiposity: are both potential therapeutic targets for tackling the metabolic syndrome? Curr Pharm Des. (2007) 13:2169–75. 10.2174/138161207781039599 17627548

